# Maximizing cardiopulmonary support as a minimally invasive platform—using peripheral parallel veno-venous and veno-arterial extracorporeal membrane oxygenation and axillary impella 5.5 as a bridge to heart–lung transplantation

**DOI:** 10.1016/j.jhlto.2026.100684

**Published:** 2026-09-14

**Authors:** Enock Adjei, Amir Teimouri Dereshgi, Kevin C. McGann, Nathan Smith, Swaroop Bommareddi, John Trahanas, Stephen DeVries, Kelly Schlendorf, Ashish S. Shah, Matthew Bacchetta, Konrad Hoetzenecker, Caitlin T. Demarest, John W. Stokes, Aaron M. Williams

**Affiliations:** a Division of Cardiac Surgery, Vanderbilt University Medical Center, Nashville, Tennessee; b Department of Cardiology, Vanderbilt University Medical Center, Nashville, Tennessee; c Department of Thoracic Surgery, Vanderbilt University Medical Center, Nashville, Tennessee; d Department of Biomedical Engineering, Vanderbilt University Medical Center, Nashville, Tennessee

**Keywords:** heart-lung transplant, awake ECMO, axillary impella, LV decompression, bridge to transplant, COVID-19 ILD

## Abstract

Combined heart–lung transplantation (HLTx) in patients with cardiopulmonary failure requiring extracorporeal membrane oxygenation (ECMO) presents a major physiologic challenge. Conventional ECMO strategies may inadequately address simultaneous refractory hypoxemia, circulatory failure, and left ventricular (LV) distension without central cannulation and sternotomy.

A 65-year-old man with severe heart failure (LVEF <10%) and rapidly progressive interstitial lung disease presented with cardiogenic shock and hypercapnic hypoxemic respiratory failure. Initial V-AV ECMO provided stabilization and allowed extubation and expedited HLTx evaluation. Progressive hypoxemia and acute kidney injury despite maximal V-AV support prompted escalation to peripheral parallel VV–VA ECMO. Echocardiography demonstrated LV distension, prompting axillary Impella 5.5 placement for decompression. The patient remained awake and extubated on parallel VV–VA ECMO and Impella 5.5 for 60 hours prior to successful heart–lung transplantation.

We report the novel application of a fully peripheral cardiopulmonary support platform as a viable bridge strategy for HLTx candidates.

Combined heart and lung transplantation (HLTx) remains uncommon, with fewer than 50 procedures performed annually in the United States.^1^ Bridge-to-transplant support for concurrent end-stage cardiac and pulmonary failure requires replacement of gas exchange and cardiac output, often with active LV decompression. VV ECMO corrects hypoxemia and hypercapnia but provides no hemodynamic support, whereas VA ECMO supports systemic perfusion but increases LV afterload and may cause LV distension. Central configurations can address these limitations but require sternotomy or thoracotomy, carry substantial bleeding risk, and may compromise transplant candidacy.^2^ We recently reported parallel peripheral VV–VA ECMO as a rescue strategy for lung transplant candidates failing single-circuit VV support.^3^ Here, we describe its first application—augmented with axillary Impella 5.5—as a bridge to combined HLTx in a patient with near-complete native cardiopulmonary failure.

A 65-year-old man with nonischemic cardiomyopathy (LVEF <10%) and COVID-19-induced pulmonary fibrosis (diagnosed 4 months earlier requiring 3 L/min supplemental oxygen at rest) maintained excellent functional status, walking approximately 4 miles daily before presentation. He presented with cardiogenic shock and refractory respiratory acidosis despite maximal ventilatory support (pH 7.11, PaCO₂ 97 mmHg). His course was further complicated by a brief ventricular arrhythmic arrest requiring 2 minutes of cardiopulmonary resuscitation.

Urgent V-AV ECMO was initiated as bridge-to-decision support via 25-Fr right femoral venous drainage, 21-Fr right internal jugular venous return, and 15-Fr right common femoral arterial return with a 6-Fr distal perfusion catheter. He was weaned from vasopressors, maintained pulsatility, and was extubated. to facilitate rehabilitation and expedited HLTx evaluation. On hospital day (HD) 2, he participated in bedside standing and ambulated within his room. He had an expediated HLTx evaluation and was approved as Status 1.

On HD 3, he developed progressive hypoxemia, with SpO2 of 75% to 80% with routine nursing bed turn—and acute kidney injury despite maximal V-AV support (total flow 6.5LPM; venous return 4.0LPM; arterial return 2.5LPM; sweep 5LPM, 100% FIO2) and high-flow nasal cannula at 40L/100% FiO2. Arterial blood gas before reconfiguration was pH 7.40, PaCO2 37 mmHg, PaO2 60 mmHg, and HCO3 23 mEq/L while the patient was receiving high-flow nasal cannula at 40 L/min with FiO2 100%). Swan-Ganz catheterization demonstrated pulmonary artery pressures of 73/40 mmHg (mean 51 mmHg) despite inhaled nitric oxide. Chest radiography demonstrated near-complete native lung failure (Figure 1C). He was transitioned to parallel VV–VA ECMO consisting of a VV circuit (21-Fr RIJ return and 25-Fr RFV drainage) and a VA circuit (23-Fr LFV drainage and 15-Fr RCFA return with distal perfusion catheter). Intraoperative echocardiography demonstrated progressive LV distension with decreased systemic pulsatility (<10 mmHg) and moderate mitral regurgitation associated with ECMO-related afterload augmentation, prompting placement of a right axillary Impella 5.5 for active LV decompression **(**Supplemental Table 3).

**Figure 1:**
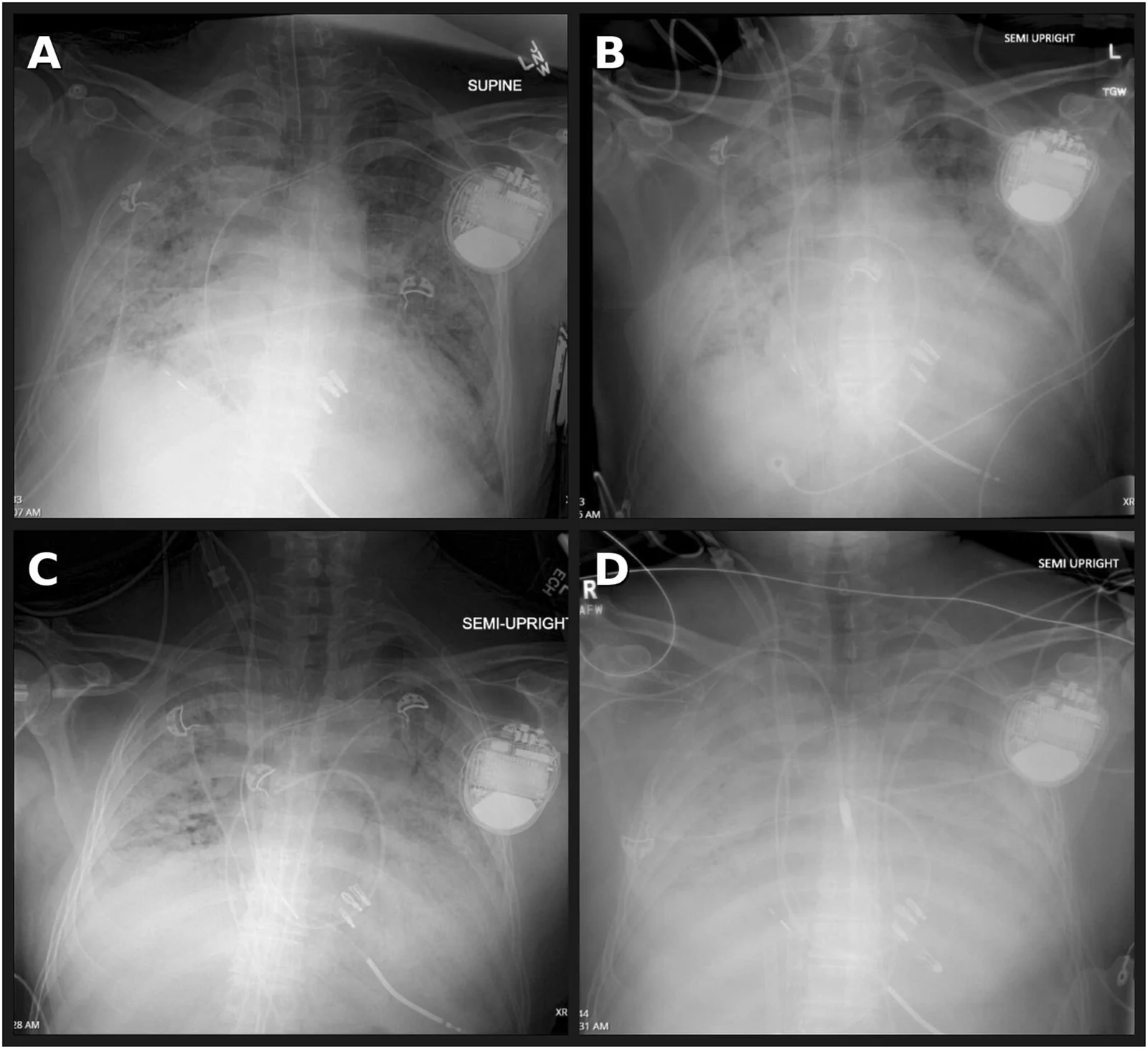
Serial chest radiographs demonstrating rapid progression of COVID-19–associated interstitial lung disease and escalation of mechanical cardiopulmonary support•**A:** HD 1: Diffuse bilateral pulmonary infiltrates consistent with severe COVID-19–associated interstitial lung disease prior to ECMO initiation•**B:** HD 2: Progressive bilateral pulmonary opacification following V-AV ECMO cannulation, with visible implantable cardioverter-defibrillator (ICD), right internal jugular (RIJ) venous return cannula, and femoral arterial return cannula•C: HD 3: Near-complete bilateral pulmonary whiteout prior to ECMO reconfiguration, reflecting progressive native lung failure despite maximal V-AV ECMO support•D: HD 4: Chest radiograph following reconfiguration to parallel VV–VA ECMO and right axillary Impella 5.5 placement, demonstrating multiple extracorporeal support devices with persistent severe bilateral pulmonary opacification

This configuration enabled independent optimization of oxygenation and hemodynamic support with VV flows of 5 L/min, VA flows of 3 L/min, and Impella support at P3–P4 generating flows of 2.0 to 2.2 L/min (Supplemental Figure 1). He remained extubated, awake, hemodynamically stable, and participated in the bed rehabilitation **(**Figure 2**).**

**Figure 2:**
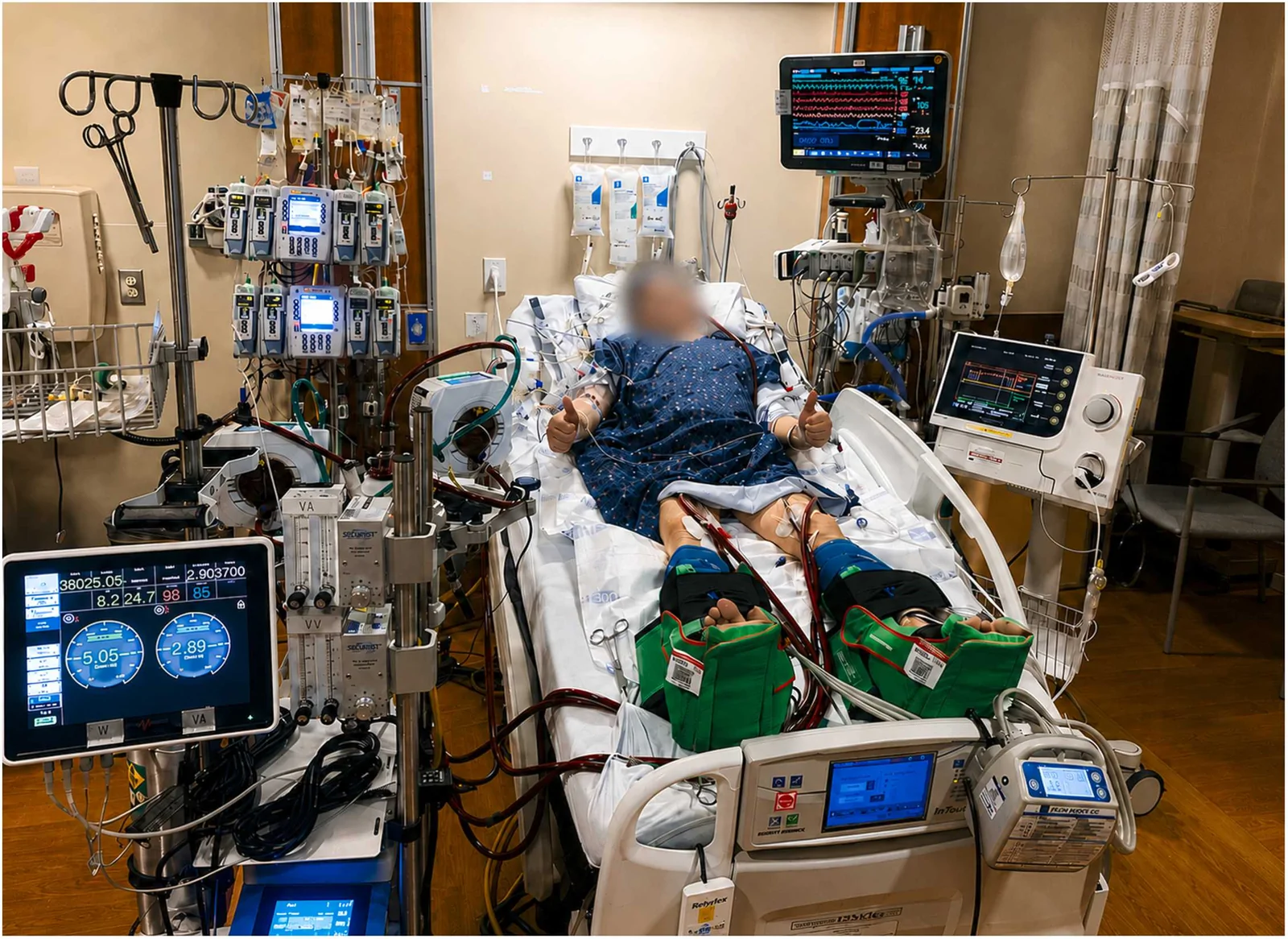
Awake and extubated patient supported with parallel VV–VA ECMO and axillary Impella 5.5 prior to combined heart–lung transplantation. Circuit 1 consisted of VV-ECMO (21-Fr right internal jugular return and 25-Fr right femoral venous drainage cannulas; flows 5.05 LPM), while Circuit 2 consisted of VA-ECMO (23-Fr left femoral venous drainage and 15-Fr right common femoral arterial return cannulas; flows 2.89 LPM). Axillary Impella 5.5 provided active left ventricular decompression. The patient remained awake, interactive, and extubated while supported on this triple-device configuration. Written informed consent was obtained from the patient for publication of this image. VV–VA ECMO, veno-venous and veno-arterial extracorporeal membrane oxygenation

A donor became available on HD 5 (Supplemental Table 1), and he underwent successful en-bloc HLTx through a clamshell incision (Supplemental Figure 2). Post-implantation echocardiography demonstrated normal biventricular function with LVEF >65%; lung mechanics and oxygenation were satisfactory without primary graft dysfunction. No postoperative MCS was required. Tracheostomy was performed on postoperative day (POD) 2 for ventilator weaning. Vasopressors and inotropes were discontinued by POD 3. He remained in the intensive care unit for 14 days while receiving continuous renal replacement therapy and was transferred to the surgical floor on POD 14. On POD 30, he developed a right groin hematoma; U/S and CT angiography showed a 6x6cm hematoma with a 4 mm right common femoral artery pseudoaneurysm at the prior VA ECMO cannulation site, requiring operative exploration and primary arterial repair followed by 2 weeks of wound VAC therapy. He was discharged to inpatient rehabilitation on POD 45 and home 1 week later. At 3 months, he was performing daily activities independently without limitation; echocardiography showed normal biventricular function (LVEF 55% to 65%) and spirometry showed FEV₁ 2.98 L and FVC 97% predicted, with no clinical or biopsy evidence of rejection or primary graft dysfunction (Supplemental Table 2**).**

This case suggests that in certain cases, parallel VV–VA ECMO combined with axillary Impella 5.5 as a fully peripheral bridge to combined HLTx. Despite near-complete native cardiopulmonary failure, the patient remained awake and engaged in rehabilitation while awaiting transplantation, extending a strategy previously described only in lung transplant candidates.^3^ This platform enabled simultaneous optimization of gas exchange, hemodynamic support, and LV decompression without sternotomy or thoracotomy while preserving transplant candidacy.

Parallel VV–VA ECMO was selected over alternative configurations for several physiologic and procedural reasons. The sport model—VA ECMO with axillary or subclavian arterial return via side graft and right internal jugular venous drainage—was considered because antegrade delivery of oxygenated blood from the axillary artery may improve upper-body oxygenation and mitigate differential hypoxemia compared with femoral arterial return.^4^, ^5^ However, this approach would have required high-flow axillary arterial return, increasing concern for axillary hyperperfusion syndrome, a recognized complication of axillary cannulation that may progress to compartment syndrome requiring fasciotomy.^6^ Furthermore, concomitant sport model support and axillary Impella 5.5 placement would have necessitated bilateral axillary cutdowns, increasing operative complexity and bleeding risk. More importantly, a single peripheral ECMO circuit cannot independently optimize gas exchange and circulatory support in patients with near-complete cardiopulmonary failure.^3^, ^7^ In contrast, parallel VV–VA ECMO permits independent titration of respiratory and hemodynamic support, enabling complete cardiopulmonary replacement while preserving a single axillary access site for Impella support and avoiding bilateral upper-extremity arterial cannulation.^3^, ^7^

Impella 5.5 placement was prompted by progressive LV distension after VA-ECMO initiation, reinforcing the importance of routine echocardiographic assessment and early LV unloading in patients with severely reduced LVEF on VA-ECMO support.^8^ VA-ECMO-induced elevation of LV end-diastolic pressure generates post-capillary pulmonary hypertension, perpetuating pulmonary edema, impaired gas exchange, and worsening mitral regurgitation. Active LV decompression with Impella 5.5 reduced LV end-diastolic pressure, lowered pulmonary venous pressures, and mitigated mitral regurgitation.

The Impella 5.5 offered advantages over alternative unloading strategies. Atrial septostomy provides indirect, non-titratable decompression with risk of paradoxical hypoxemia; intra-aortic balloon counterpulsation is often insufficient in near-absent LV contractility; and surgical LV venting requires thoracotomy or sternotomy under full anticoagulation, which carry a *substantial perioperative morbidity, and* potentially compromising the operative field for the anticipated clamshell HLTx incision and threatening the awake state essential for transplant candidacy. In contrast, axillary Impella 5.5 provides titratable active decompression without cardiotomy or thoracotomy while permitting rehabilitation and mobilization.^9^

This strategy is not without risks, particularly those inherent to multisite peripheral cannulation. In this case, a common femoral artery pseudoaneurysm at the prior VA-ECMO cannulation site required operative repair, highlighting the importance of meticulous vascular surveillance and decannulation management when employing this approach.

Parallel VV–VA ECMO with axillary Impella 5.5 is a novel, sternotomy- and thoracotomy-free platform that permits independent optimization of respiratory and circulatory support, enabling near-complete cardiopulmonary replacement as a bridge to combined HLTx. This approach preserves an awake, extubated state and rehabilitation potential while awaiting transplantation. The case also underscores the importance of physiologic rather than chronologic age in candidate selection, as exceptional premorbid functional capacity may permit favorable outcomes despite advanced age. Further experience is needed to define the role of this strategy in heart–lung transplantation.

## Ethics statement

This transplantation was performed in accordance with the Declaration of Istanbul and UNOS/OPTN regulatory guidelines governing deceased donor organ allocation in the United States.

## Funding statement

None.

## Consent

Written informed consent was obtained from the patient for publication of clinical photograph.

## Financial disclosures

None.

## Declaration of generative AI and AI-assisted technologies in the writing process

We declare that no AI or AI-assisted technologies were used in the writing or editing of this manuscript.

## Declaration of Competing Interest

We declare that we have no competing interests relevant to this work. No author has any financial or personal relationships that could inappropriately influence or bias the content of this manuscript.

## Appendix A Supplementary material

Supplementary material

**Supplemental Figure 1:** Peripheral parallel VV–VA ECMO configuration. Circuit 1 (VV): 21-Fr RIJ return, 25-Fr RFV drainage (5.0 LPM, FiO_2_ 100%). Circuit 2 (VA): 23-Fr LFV drainage, 15-Fr right CFA return, 6-Fr right SFA DPC (2.8–3.0 LPM). Impella 5.5 inserted via right axillary artery side graft, coursing retrograde through the subclavian artery and aortic arch, across the aortic valve into the LV

**Supplemental Figure 2:** Explanted native cardiopulmonary organs and donor heart–lung allograft. (A) Explanted native heart and lungs demonstrating severe diffuse COVID-19–associated fibrotic lung disease with markedly abnormal, consolidated parenchyma and enlarged cardiomyopathic heart. (B) Donor en-bloc heart–lung allograft prior to implantation, demonstrating preserved donor lung parenchyma and cardiac allograft anatomy

## Footnotes

Appendix A Supplementary data associated with this article can be found in the online version at doi:10.1016/j.jhlto.2026.100684.
