# Supplementary material for: Maximizing cardiopulmonary support as a minimally invasive platform—using peripheral parallel veno-venous and veno-arterial extracorporeal membrane oxygenation and axillary impella 5.5 as a bridge to heart–lung transplantation
Source: JHLT Open. 2026 Sep 14;14:100684. doi: 10.1016/j.jhlto.2026.100684 (PMC13659484; doi:10.1016/j.jhlto.2026.100684)
Supplement: Supplementary file 1 [file mmc1.docx]

**Supplemental Table 1. Baseline Donor and Recipient Characteristics.**

| **Variables** | **Donor** |
| --- | --- |
| Age (years) | 45 |
| Sex (Male/Female) | Female |
| BMI (kg/m2) | 34.85 |
| Cause of Death | Head trauma |
| Donor CPR duration | - |
| Diabetes mellitus | No |
| Hypertension | No |
| Prior MI | No |
| Chest Trauma | No |
| Smoking History | No |
| HCV Status | Negative |
| LVEF | 55-65% |
|  |  |
| **Variables** | **Recipient** |
| Age (years) | 65 |
| Sex (Male/Female) | Male |
| BMI (kg/m2) | 23.03 |
| PHM Ratio | 0.97 |
| Heart failure cause | NICM |
| Diabetes mellitus | No |
| Hypertension | No |
| Chronic Kidney Disease | Yes |
| Waitlist status | 1 |
| Perioperative MCS | Yes (parallel VV-VA ECMO and impella 5.5) |
| Multiorgan Transplant | Yes (Heart-Lung) |
| Prior Sternotomy | No |

BMI: Body mass index, CPR: Cardiopulmonary resuscitation, MI: Myocardial infarction, HCV: Hepatitis C virus, LVEF: Left ventricular ejection fraction, PHM: predicted heart mass, NICM: non-ischemic cardiomyopathy, MCS: Mechanical circulatory support, LVAD: Left ventricular assist device.

**Supplemental Table 2. Intraoperative and Postoperative Outcomes.**

| **Variables** | **Recipient** |
| --- | --- |
| CP Bypass length | 222 mins |
| Donor Cross Clamp On To Recipient Cross Clamp Off | 313 mins |
| Post-bypass LVEF | >55% |
| Post-bypass RV function | Normal function |
| Postoperative MCS | NO |
| Cardiac Severe PGD (within 24 h)** | NO |
| Pulmonary PGD 3 (24 and 72 hours) | NO |
| LVEF (POD: 7) | Normal |
| RV function (POD:7) | Normal |
| Need for renal replacement therapy or hemodialysis | Yes |
| Post operative DVTs: Non-occlusive RIJ and LIJ deep vein thrombosis on routine U/S on POD 7, (managed with anticoagulation) | Yes |
| Vascular complications | Yes; post operative right common femoral artery pseudoaneurysm on POD 30, requiring vascular intervention. |
| Endomyocardial Biopsy (to be determined) | ACR/AMR: N/A |
| Lung Biopsy (3 months post-transplant) | Negative for ACR |
| Renal function (3 months post-transplant) | Off dialysis with slow renal recovery, Creatinine 2.58 mg/dL and eGFR of 27 from nadir of 16. |

Cardiopulmonary MCS — Mechanical circulatory support PGD — Primary graft dysfunction POD — Postoperative day LVEF — Left ventricular ejection fraction RV — Right ventricle DVT — Deep vein thrombosis RIJ — Right internal jugular LIJ — Left internal jugular U/S — Ultrasound ACR — Acute cellular rejection AMR — Antibody-mediated rejection N/A — Not applicable

**Supplemental Table 3. Patient Characteristics, Hemodynamic Profile, and Extracorporeal Support Parameters**

| **PART A — Baseline and Pre-Admission Characteristics** | |
| --- | --- |
| **Variable** | **Value** |
| **Demographics** | |
| Age (years) | 65 |
| Sex | Male |
| Body mass index (kg/m²) | 23.03 |
| Body surface area (m²) | 1.86 |
| **Diagnosis and Disease Duration** | |
| Duration of nonischemic cardiomyopathy (years) | ~1 |
| Duration from COVID ILD diagnosis to acute decompensation (months) | 1 |
| **Comorbidities** | |
| Diabetes mellitus | No |
| Hypertension | No |
| Pre-existing renal insufficiency | Yes |
| Serum creatinine at admission (mg/dL) | 1.40 |
| eGFR at admission (mL/min/1.73 m²) | 52 |
| **Baseline Cardiopulmonary Profile (Prior RHC, 2025)†** | |
| LV ejection fraction (%) | 10–15 |
| Baseline RV function | Normal |
| Pulmonary artery pressure (mmHg) | 57/23 (mean 34) |
| Fick cardiac output (L/min) | 3.13 |
| Pre-existing pulmonary hypertension | Yes |
| **Admission Status** | |
| Admission arterial blood gas (pH / PaCO₂ mmHg) | 7.11 / 97 |
| Admission lactate (mmol/L) | 3.2 |
| Admission RV function (bedside POCUS) | Moderate-to-severe dysfunction |

| **PART B — ECMO Parameters and Hemodynamics Before and After Reconfiguration** | | |
| --- | --- | --- |
| **Variable** | **V-AV ECMO**  **(Pre-reconfiguration)** | **Parallel VV–VA ECMO + Impella 5.5** |
| **ECMO Settings** | | |
| Total ECMO flow (L/min) | 6.5 | VV: 5.0 / VA: 3.0 |
| Venous return flow (L/min) | 4.0 | 5.0 |
| Arterial return flow (L/min) | 2.5 | 3.0 |
| Impella flow (L/min) | — | 2.0–2.2 (P3–P4) |
| VV circuit sweep gas (L/min) | 5.0 | 4.0 |
| VA circuit sweep gas (L/min) | — | 2.0 |
| Circuit FiO₂ | 100% | 100% |
| **Respiratory Support** | | |
| Supplemental oxygen | HFNC 40 L/min, FiO₂ 100% | 6 L/min nasal cannula |
| SpO₂ nadir with routine bed turns | 75–80% | >95% |
| **Arterial Blood Gas** | | |
| pH | 7.40 | 7.43 |
| PaCO₂ (mmHg) | 37 | 35 |
| PaO₂ (mmHg) | 60 | 180 |
| HCO₃ (mEq/L) | 23 | 24 |
| **Hemodynamics** | | |
| Pulmonary artery pressure (mmHg) | 73/40 (mean 51) | 50s/30s (mean 35–40)‡ |
| Inhaled nitric oxide | Yes | Yes |
| Vasopressors | Yes (norepinephrine 5-10mcg) | Weaned off |
| Creatinine mg/dL | 2.89 | 2.07 |
| **Echocardiography*** | | |
| LVEDD / LVESD (cm) | 8.47 / 7.98 | — |
| LV ejection fraction (%) | 10 | 5–10* |
| Mitral regurgitation | Moderate | Mild-to-moderate* |
| RV function | Moderate-to-severe dysfunction | Moderate-to-severe dysfunction* |
| TAPSE (cm) | 1.37 | — |

ILD, Interstitial lung disease; RHC, Right heart catheterization; POCUS, Point-of-care ultrasound; ECMO, Extracorporeal membrane oxygenation; VV, Veno-venous; VA, Veno-arterial; V-AV, Veno-arteriovenous; HFNC, High-flow nasal cannula; ABG, Arterial blood gas; LVEDD, Left ventricular end-diastolic diameter; LVESD, Left ventricular end-systolic diameter; LVEF, Left ventricular ejection fraction; TAPSE, Tricuspid annular plane systolic excursion; RV, Right ventricle; PAP, Pulmonary artery pressure; iNO, Inhaled nitric oxide.

† Baseline cardiopulmonary profile obtained from prior right heart catheterization performed in 2025, prior to acute decompensation.

* Post-reconfiguration echocardiography was a focused study performed primarily for Impella positioning confirmation; comprehensive hemodynamic assessment was not the clinical objective given the bridge-to-transplant trajectory. The apparent reduction in LVEF (10% → 5–10%) reflects ongoing VA-ECMO afterload augmentation at the time of imaging rather than true deterioration. Improvement in mitral regurgitation from moderate to mild-to-moderate represents the earliest echocardiographic marker of effective LV unloading.

‡ Measured after reconfiguration to parallel VV–VA ECMO and Impella 5.5 placement; formal repeat right heart catheterization was not performed during the bridge interval
